# Nailfold capillaroscopy in 430 patients with rheumatoid arthritis

**DOI:** 10.22088/cjim.8.4.269

**Published:** 2017

**Authors:** Alireza Rajaei, Pooneh Dehghan, Ali Amiri

**Affiliations:** 1 Department of Rheumatology, Loghman Hospital, Shahid Beheshti University of Medical Sciences, Tehran, Iran.; 2 Department of Radiology, Taleghani Hospital, Shahid Beheshti University of Medical Sciences, Tehran, Iran.; 3 Shahid Beheshti University of Medical Sciences, Tehran, Iran.

**Keywords:** Rheumatoid arthritis, Nailfold video capillaroscopy, Scleroderma pattern.

## Abstract

**Background:**

Microvascular changes are one of the first obvious steps in numerous inflammatory diseases such as rheumatoid arthritis (RA). Nailfold video capillaroscopy (NFC) is an easy, reliable and safe method for evaluating peripheral microangiopathy. The objective of this study was to examine nailfold microcirculation in RA patients, assess morphological and structural changes quantitatively and qualitatively, and recognize useful changes.

**Methods:**

A total of 430 patients diagnosed with RA were examined in a period of 4 years. NFC was performed on all fingers of both hands in each patient. Different parameters indicating microvascular changes were detected and analyzed; such as microvascular architecture, capillary distribution disturbances, capillary morphology, capillary density, efferent/afferent limb ratio, subpapillary venular plexus and morphological abnormalities. The obtained results were categorized into normal pattern, nonspecific morphological abnormality and scleroderma pattern.

**Results:**

The mean age of participants was 51.03±14.54 (19-87 years) that consisted of 359 females and 71 males. Based on the findings, angiogenesis (74.7%) was the most pathological condition observed after tortuosity (99.5%). 7.2% and 20.9% of patients were categorized into normal and scleroderma pattern group, respectively. Among morphological abnormalities, angiogenesis, isolated enlarged loop, irregular enlarged loop and architectural derangement were significantly more frequent in scleroderma than normal pattern (p<0.001).

**Conclusion:**

NFC may play an important role in monitoring RA disease and patients’ follow-up. Therefore, in our opinion it could be considered in the course and follow-up of rheumatoid arthritis.

**R**heumatoid arthritis (RA) is a chronic and multisystem autoimmune disease characterized by persistent and symmetrical polyarthritis, associated with extra articular involvement in some patients. RA has a worldwide prevalence of 1%. It can affect every age group, but it occurs more commonly in the 4th or the 5th decades of life and women are about 3 times more susceptible than men (1-3). Altered vascular environment is a significant aspect of RA’s pathophysiology (1). Rheological and morphological damages of microcirculation have been reported in RA patients. These damages are reported to be the result of capillary permeability impairments and alterations in endothelium connections with extracellular matrix, which cause abnormal capillary shapes and structures in the dermal papillae (4, 5). Video capillaroscopy can simply recognize peripheral microangiopathy, even in the early stages of the disease (6). Nailfold video capillaroscopy (NFC) is a simple, noninvasive, inexpensive and highly sensitive method for morphological study of papillary dermal capillaries.

This technique has been widely been used in many rheumatologic disorders such as systemic sclerosis, Raynaud’s phenomenon and connective tissue diseases (7). Besides in patients with systemic disease, capillaroscopy is useful for predicting the possibility of developing digital ulcers and visceral complications (8).

Different devices such as microscope, dermatoscope, ophtalmoscope and videocapillaroscope are used for the evaluation of microcirculation, but videocapillaroscope is an upgraded method for this purpose and it is considered as a gold standard method for non-invasive examination of microcirculation. It contains a digital video camera and a microscope and in comparison with other optical devices, it provides a significantly better magnification (9, 10). NFC has many advantages such as allowing the quantification of any loop abnormalities, measure individual capillaries and capable of storing images for later analysis or reanalysis (11). 

For the evaluation of capillaroscopic pattern, the combination of morphological and numerical characteristics of the image is required, and obviously it cannot be evaluated based on a single parameter (9). Using NFC, the subsequent factors measured for the evaluation of pathologic capillaroscopic changes are as follows: presence of giant and enlarged capillaries, disorganization of the vascular arrangement, hemorrhages, ramified/bushy capillaries and loss of capillaries (6). No specific changes have been reported in the capillaroscopic examination of RA patients. It could be very important for the therapy of these patients to establish the diagnosis of vascular pathology in earlier stages with a reliable method like capillaroscopy. Hence, the aim of this study was to examine nailfold microcirculation in this population, assess morphological and structural changes quantitatively and qualitatively, and recognize useful changes.

## Methods

In this cross-sectional study, NFC findings of 430 RA patients have been collected in Resalat hospital (a private general hospital in Tehran, Iran) from October 2011 to June 2015. All patients were between 19 and 87 years of age. Patients were included in this study if they met the following criteria: rheumatoid arthritis diagnosed according to American College of Rheumatology criteria (ACR) (12) for at least one year, and excluded if they had history of smoking, other autoimmune diseases, acute infection, diabetes mellitus, renal insufficiency, hypertension, dyslipidemia or drugs affecting the cardiovascular system. Written informed consent was obtained from all the participants and the study protocol was approved by Research and Ethics Committee of Tehran Resalat Hospital. In standard conditions, all participants were informed about the test and asked to avoid caffeine consumption for at least 12 hours before the test. 

At first the patients were asked to remain in semi sitting position with hands placed at the same level as heart in an adapted room (with its temperature about 22-25◦C) for at least 15 minutes to balance the body temperature with the environment. Due to morphology variations in RA from a nailfold to another, the periungual zone of ten fingers were examined. 

The test was done using a video capillaroscope (D1 videocap, Vedica, SRL, DC, Milan, Italy, 2011), after placing a drop of cedar or olive oil on the nailfold bed for better transparency of the capillaries. The optical microscope was linked to a computer with image analysis software (Videocap; DS MediGroup, Milan, Italy) and a digital camera. All capillaroscopy images were evaluated by an expert rheumatologist. In periungual region, projection of capillary loops into the dermal papillae, allows a longitudinal view of its three sections (transition, afferent and efferent) parallelly organized to the skin (7). 

Subsequent capillaroscopic factors were measured by an expert rheumatologist for each image: capillary density (the entire number of erythrocyte-perfused capillaries per square millimeter of skin), microvascular architecture, capillary distribution (morphology and numbers), morphological abnormalities (Raynaud loops, mega capillaries and bushy capillaries), efferent/afferent limb ratio and subpapillary venular plexus (based on Wertheimer’s criteria adjusted by Terreri *et al*.) (13, 14).

Results were categorized as scleroderma pattern, normal and non-specific morphological abnormalities. Conventionally, if an abnormal finding was observed in at least two fingernails of a patient, it was considered significant. According to a study by Cutolo et al., patients were categorized into two different groups (6, 7). Patients with hairpin-shaped capillaries homogeneously distributed (comb-like structure), and a density of average 10 capillaries per mm (between 9 and 14) or only one abnormal factor (non-specific morphological abnormalities), were defined as normal capillaroscopic pattern. Patients who had two or more certainly abnormal morphological patterns in more than one evaluated fingernails, were defined as scleroderma pattern. All variables were analyzed qualitatively and quantitatively using SPSS Version 23. For qualitative variables, chi-square test was used. If the p-value is less than 0.05, results are considered statistically significant.

## Results

**Figure 1:**
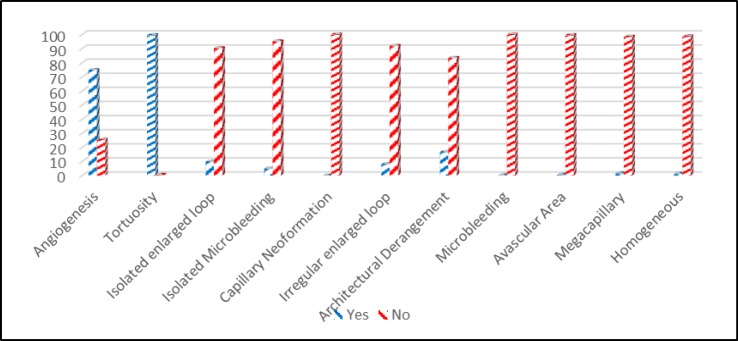
Morphological abnormalities shown in capillaroscopy of RA patients

Among the 430 patients with RA who met the inclusion criteria, 71(16.5%) were males and 359 (83.5%) were females. The patient’s mean age at the time of capillaroscopy was 51.03±14.54 years. Normal pattern was observed in 31 (7.2%), scleroderma pattern in 90 (20.9%) and nonspecific morphological abnormality in 309 (71.8%) patients. In more than 97% of patients, capillary distribution, capillary morphology, capillary density, subpapillary venular plexus visibility and efferent/afferent ratio were normal but microvascular architecture was abnormal in 22.1% of patients (table 1). Tortuosity and angiogenesis were the most common morphological abnormalities present in 428 (99.5%) and 321 (74.7%) patients, respectively. Details of the morphological abnormalities in all patients are shown in figure 1.

**Table 1:** Capillaroscopic Findings of 430 Rheumatoid arthritis patient

	**Total** **N(%)**
**Microvascular architecture**	
Normal Abnormal Undetectable	329 (76.5) 95 (22.1) 6 (1.4)
**Capillary distribution**	
Regular Irregular	426 (99.1) 4 (0.9)
**Capillary morphology**	
Homogeneous Nonhomogeneous	425 (98.8) 5 (1.1)
**Capillary density**	
Normal Decreased	421 (97.9) 9 (2.1)
**Sub papillary venular plexus**	
Visible Invisible	425 (98.8) 5 (1.1)
**Efferent/Afferent ratio**	
Normal Increased	424 (98.6) 6 (1.4)

Of all the patients with scleroderma pattern, 74 (82.2%) were females. Among morphological abnormalities in patients with scleroderma pattern, capillary tortuosity, angiogenesis and isolated enlarged loop were the most frequent findings, respectively. Altered microvascular architecture was significantly more frequent in patients with scleroderma pattern than patients with normal pattern (p<0.05). In morphological abnormalities, angiogenesis, isolated enlarged loop, irregular enlarged loop and architectural derangement were significantly higher in patients with scleroderma pattern in comparison to patients with normal pattern (p<0.05).Tables 2 and table 3 emonstrate the details. 

**Table 2:** Comparison of capillaroscopic variables in RA patients with normal and scleroderma pattern

	**Normal pattern** **n (%)**	**Scleroderma pattern ** **n (%)**	**P-value**
**Microvascular architecture**			
Normal Abnormal Undetectable	31 (100) 0 0	48 (53.3) 41 (45.6) 1 (1.1)	0.000
**Capillary distribution**			
Regular Irregular	31 (100) 0	88 (97.8) 2 (2.2)	0.403
**Capillary morphology**			
Homogeneous Nonhomogeneous	31 (100) 0	88 (97.8) 2 (2.2)	0.403
**Capillary density**			
Normal Decreased	31 (100) 0	85 (94.4) 5 (5.6)	0.180
**Efferent/Afferent limb ratio**			
Normal Increased	31 (100) 0	86 (95.6) 4 (4.4)	0.233
**Subpapillary venular plexus**			
Visible Invisible	29 (93.5) 2 (6.5)	89 (98.9) 1 (1.1)	0.099
**Gender**			
Male Female	4 (12.9) 27 (87.1)	16 (17.8) 74 (82.2)	0.529

**Table 3:** Comparison of architectural abnormalities in RA patients with normal and scleroderma pattern

**Architectural abnormality**		**Normal Pattern** **n (%)**	**Scleroderma pattern** **n(%)**	**P-value**
Angiogenesis	Yes No	3 (9.7) 28 (90.3)	81 (90) 9 (10)	0.000
Tortuosity	Yes No	30 (96.8) 1 (3.2)	90 (100) 0 (0)	0.087
Isolated enlarged loop	Yes No	0 (0) 31 (100)	31 (34.4) 59 (65.6)	0.000
Isolated microbleeding	Yes No	1 (3.2) 30 (96.8)	15 (16.7) 75 (83.3)	0.057
Capillary neoformation	Yes No	0 (0) 31 (100)	0 (0) 90 (100)	_
Irregular enlarged loop	Yes No	0 (0) 31 (100)	27 (30) 63 (70)	0.001
Architectural derangement	Yes No	0 (0) 31 (100)	28 (31.1) 62 (68.9)	0.000
Microbleeding	Yes No	1 (3.2) 30 (96.8)	0 (0) 90 (100)	0.087
Avascular area	Yes No	0 (0) 31 (100)	2 (2.2) 88 (97.8)	0.403
Mega capillary	Yes No	0 (0) 31 (100)	5 (5.6) 85 (94.4)	0.180
Homogeneous enlarged loop	Yes No	0 (0) 31 (100)	4 (4.4) 86 (95.6)	0.233

Although the patients with scleroderma pattern were slightly older than those with normal pattern, this difference was not statistically significant (p=0.405). Also, there was no correlation between patient’s gender and their capillaroscopic patterns (normal or scleroderma). Table 4 shows a comparison of all microvascular abnormalities between both genders; accordingly, angiogenesis, isolated micro bleeding, architectural derangement and homogenous enlarged loop were statistically significant between men and women (p<0.05).

**Table 4:** Comparison of microvascular abnormalities between genders

**Variables**	**Male (%)**	**Female (%)**	**P-value**
Altered microvascular architecture	15.5	23.4	0.167
Nonhomogeneous capillary distribution	0	1.1	0.372
Nonhomogeneous capillary morphology	0	1.4	0.317
Reduced capillary density	1.4	2.2	0.659
Increased efferent/afferent limb ratio	1.4	1.4	0.992
Invisible sub papillary venular plexus	1.4	1.1	0.833
Angiogenesis	84.5	72.7	0.037
Tortuosity	100	99.4	0.528
Isolated enlarged loop	15.5	8.6	0.075
Isolated microbleeding	0	5.8	0.037
Capillary neoformation	0	0	_
Irregular enlarged loop	5.6	8.6	0.398
Architectural derangement	8.5	18.4	0.041
Microbleeding	0	0.3	0.656
Avascular area	0	0.6	0.528
Mega capillary	4.2	1.1	0.058
Homogeneous enlarged loop	4.2	0.8	0.026

## Discussion

In this study, we evaluated nailfold capillaroscopic abnormalities of 430 patients with rheumatoid arthritis and showed their pathological changes. According to these findings, capillaroscopic morphological abnormalities were significant in micro circulation of RA patients. Rheumatoid arthritis is a chronic inflammatory autoimmune disease along with an extensive range of extra-articular involvement. Some studies highlighted that endothelial cells have an inflammatory role in the disease process and cause systemic organ manifestations (either by new blood vessels proliferation or by overexpression of inflammatory mediators) (15). 

Clinical manifestations of RA (especially the seronegative type) are so similar to psoriatic arthritis that cause difficult differentiation between these diseases (16). The presence of a specific capillaroscopic pattern in RA patients is a matter of argument. Previously, microvascular permeability changes in RA were shown in 1994 by Hachulla et al. to support the presence of microangiopathy (4). In addition, microvascular dysfunction and alterations of the normal blood stream velocity in RA patients has been reported by Meyer et al. (17). In 1968, Schumacher et al. deprived the existence of specific capillaroscopic changes in RA (18). However, some studies found nonspecific capillaroscopic changes. According to these studies, presence of elongated and thin palisade capillaries, capillary low density, microhemorrages and subpapillary venular plexus visibility are reported in RA patients. Although they seem to be some characteristics of RA, they do not seem to be pathognomonic (19-21). 

We observed a high frequency of capillary morphologic changes, especially increased angiogenesis and capillary tortuosity. Altomonte et al. also found that tortuosity and elongated loops are the main changes in patients with RA (22). Of all our patients, 20.9% of 430 had different degrees of scleroderma pattern. In comparison to our results, S.N. Lambova et al. and S Pavlov-Dolijanovic et al., showed lesser frequency of scleroderma like pattern (about 14.5% of 62 patients and 7% of 106, respectively) (10, 23). These studies were performed on a small population of RA patients, also the latter only included RA patients with Raynaud’s phenomenon. All these can be the reason of differences between our results and theirs. The most frequent capillaroscopic findings were tortuosity in 99.5% and prominent subpapillary plexus in 98.8%. Lin et al. also reported that the most frequent findings in RA are tortuosity and elongated capillaries, and showed that subpapillary venular plexus (especially in positive antinuclear antibody patients) is another typical finding in RA (24).

We showed that 9.8% of all RA patients has isolated enlarged loops. Among them, 73.8% showed scleroderma pattern and none of them categorized as normal pattern. Dario Graceffa et al. reported that in RA patients, measures of the arterial limb diameter (afferent branch), venous limb diameter (efferent branch), and loop diameter were significantly more than the control group (25).

The major strength of our study was showing the capabilities of nail fold capillaroscopy; a useful technique for revealing microvascular changes in RA. In addition, a study with this size had not been performed on RA patients in Iran and it could provide a useful data source for comparison with others. Besides having beneficial strengths, our study was not without limitations. We did not consider the level of rheumatoid factor, presence of antinuclear antibody or existence of Raynaud phenomenon in participants. Moreover, the relationship between abnormal parameters and severity or duration of disease have not been assessed.

In conclusion, results from the present study state that rheumatoid arthritis has an influence on morphology and structure of microvascular circulation. This effect may predispose to occurrence of some complications. In our opinion, capillaroscopy is a very beneficial technique for analysis of microcirculation in these patients and the findings could be useful in the course and follow-up. Likewise, further studies are required to evaluate the relationship between these findings and disease severity or duration.

## References

[1] Paleolog EM (2009). The vasculature in rheumatoid arthritis: cause or consequence?. Int J Exp Pathol.

[2] Heidari B (2011). Rheumatoid arthritis: early diagnosis and treatment outcomes. Caspian J Intern Med.

[3] Dhawan SS, Quyyumi AA (2008). Rheumatoid arthritis and cardiovascular disease. Curr Atheroscler Rep.

[4] Hachulla E, Perez-Cousin M, Flipo RM (1994). Increased capillary permeability in systemic rheumatoid vasculitis: detection by dynamic fluorescence nailfold videomicroscopy. J Rheumatol.

[5] Zaric D, Worm AM, Stahl D, Clemmensen OJ (1981). Capillary microscopy of the nailfold in psoriatic and rheumatoid arthritis. Scand J Rheumatol.

[6] Cutolo M, Pizzorni C, Tuccio M (2004). Nailfold videocapillaroscopic patterns and serum autoantibodies in systemic sclerosis. Rheumatology (Oxford).

[7] Cutolo M, Sulli A, Smith V (2013). How to perform and interpret capillaroscopy. Best Pract Res Clin Rheumatol.

[8] Rajaei A, Dehghan P, Farahani Z (2015). Nailfold Capillaroscopy findings in diabetic patients (a pilot cross-sectional study). O J Pathology.

[9] Ingegnoli F, Gualtierotti R, Lubatti C (2013). Nailfold capillary patterns in healthy subjects: a real issue in capillaroscopy. Microvasc Res.

[10] Lambova SN, Müller-Ladner U (2012). Capillaroscopic pattern in inflammatory arthritis. Microvasc Res.

[11] Ingegnoli F, Gualtierotti R, Lubatti C (2009). Feasibility of different capillaroscopic measures for identifying nailfold microvascular alterations. Semin Arthritis Rheum.

[12] Arnett FC, Edworthy SM, Bloch DA (1988). The American Rheumatism Association 1987 revised criteria for the classification of rheumatoid arthritis. Arthritis Rheum.

[13] Terreri MT, Andrade LE, Puccinelli ML, Hilário MO, Goldenberg J (1999). Nail fold capillaroscopy: Normal findings in childrenand adolescents. Semin Arthritis Rheum.

[14] Wertheimer N, Werthelmer M (1955). Capillary structure: its relation to psychiatric diagnosis and morphology. J Nerv Ment Dis.

[15] Kuryliszyn-Moskal A, Klimiuk PA, Sierakowski S, Ciolkiewicz M (2006). A study on vascular endothelial growth factor and endothelin-1 in patients with extra-articular involvement of rheumatoid arthritis. Clin Rheumatol.

[16] Rajaeia A, Dehghan P (2016). Microvascular changes in patients with psoriatic arthritis as shown in nail fold capillaroscopy. Int J Sci Basic Appl Res.

[17] Meyer MF, Schmidt O, Hellmich B (2007). Microvascular dysfunction in rheumatoid arthritis assessed by laser doppler anemometry: relationship to soluble adhesion molecules and extraarticular manifestations. Rheumatol Int.

[18] Schumacher HR, Ligot PN, Barry PE (1968). Conjunctival and nailfold microcirculation in patients with rheumatoid arthritis and normal subjects. Acta Rheumatol Scand.

[19] Scardina GA, Messina P (2006). Microvascular abnormalities in patients with rheumatoid arthritis. Ann Anat.

[20] Souza EJ, Kayser C (2015). Nailfold capillaroscopy: relevance to the practice of rheumatology. Rev Bras Reumatol.

[21] Faggioli P, Tamburello A, Sciascera A, Gilardi AG, Mazzonel A (2015). Nailfold videocapillaroscopy in internal medicine. Ital J Med.

[22] Altomonte L, Zoli A, Galossi A (1994). Microvascular capillaroscopic abnormalities in rheumatoid arthritis patients. Clin Exp Rheumatol.

[23] Pavlov-Dolijanovic S, Damjanov NS, Stojanovic RM, Vujasinovic Stupar NZ, Stanisavljevic DM (2012). Scleroderma pattern of nailfold capillary changes as predictive value for the development of a connective tissue disease: a follow-up study of 3,029 patients with primary Raynaud’s phenomenon. Rheumatol Int.

[24] Lin KM, Cheng TT, Chen CJ (2009). Clinical applications of nailfold capillaroscopy in different rheumatic diseases. J Intern Med.

[25] Graceffa D, Amorosi B, Maiani E (2013). Capillaroscopy in psoriatic and rheumatoid arthritis: a useful tool for differential diagnosis. Arthritis.

